# Open Tracheostomy after Aborted Percutaneous Approach due to Tracheoscopy Revealing Occult Tracheal Wall Ulcer

**DOI:** 10.1155/2013/190818

**Published:** 2013-07-17

**Authors:** John Schweiger, Collin Sprenker, Devanand Mangar, Rachel Karlnoski, Naga Pullakhandam, Enrico M. Camporesi

**Affiliations:** ^1^Florida Gulf to Bay Anesthesiology Associates, LLC, Tampa, FL 33606, USA; ^2^University of South Florida, Department of Surgery, Tampa, FL 33606, USA; ^3^Florida Hospital, Orlando, FL 32804, USA

## Abstract

Tracheostomy is a common procedure for intensive care patients requiring prolonged mechanical ventilation. In this case report, we describe a 78-year-old female patient admitted for an aneurysm of the cerebral anterior communicating artery. Following immediate endovascular coiling, she remained ventilated and was transferred to the neurological intensive care unit. On postoperative day ten, a percutaneous tracheostomy (PCT) was requested; however, a large ulcer or possible tracheoesophageal fistula was identified on the posterior tracheal wall following bronchoscopic assessment of the trachea. Therefore, the requested PCT procedure was aborted. An open tracheostomy in the operating room was completed; however, due to the position and depth of the ulcer, a reinforced endotracheal tube (ETT) was placed via the tracheostomy. Four days later, the reinforced ETT was replaced with a Shiley distal extended tracheostomy tube to bypass the ulceration. Careful inspection and evaluation of the tracheostomy site before PCT prevented a potentially life-threatening issue in our patient.

## 1. Introduction

Tracheostomy is a routine procedure for critically ill patients. The percutaneous tracheostomy (PCT) approach has been shown to be safer and is the preferred method compared to the open technique [1, 2]. However, there are still some instances in which open tracheostomies are the necessary method.

The technique and equipment of PCT have significantly evolved since the first description of the percutaneous dilatational report described by Ciaglia et al. [3]. Bronchoscopic guidance with a fiberoptic endoscope during PCT allows direct visualization for tracheal tube positioning placement and control of the entire PCT [4–6]. Lower complication and infection rates with PCT procedures performed under bronchoscopic guidance versus “blind” PCT have been demonstrated [5, 7, 8]. Bronchoscopic guidance may also prevent iatrogenic damage to the posterior tracheal wall. Despite this, recent reports have concluded that routine bronchoscopy is not recommended prior to PCT [9, 10].

This case presents a sequence of altered clinical decisions after bronchoscopic visualization of the trachea revealed a large unexpected posterior wall ulcer before PCT. We aim to emphasize the importance of routine bronchoscopy before a tracheostomy, to assess the tracheal wall and to verify correct placement.

## 2. Case Report

A 78-year-old female with past medical history of hypertension, chronic obstructive pulmonary disease, gastroesophageal reflux disease, dyslipidemia, and panic disorder was admitted to an outside hospital after falling in her home. Cranial CT revealed an intraparenchymal hemorrhage with intraventricular extension within the right frontal segment of her brain. A subsequent CT angiogram revealed an aneurysm of the anterior communicating artery. The patient was transferred to our hospital for further care and evaluation.

The hemorrhage was categorized as world federation of neurosurgeons (WFNS) grade 2 and Fisher grade 4. Following admission to the neurological intensive care unit (ICU), the patient underwent endovascular coiling of the aneurysm after general endotracheal anesthesia was initiated. An attempted intraoperative nasogastric tube placement was traumatic, resulting in bleeding from bilateral nasal passages and blood pooling into the oropharynx. An orogastric tube was then placed successfully. After the procedure, on postoperative day 1, the patient remained ventilated and was transferred to the ICU where the ear, nose, and throat (ENT) service repaired the oropharyngeal trauma.

Our critical care service was initially consulted on hospital admission day five for acute respiratory failure secondary to a new left lower lobe atelectasis and small left pleural effusions as indicated from a chest X-ray. Upon initial bronchoscopy inspection, the distal tip of the ETT was found to be 1 cm above the carina; therefore, the cuff was deflated, and the ETT was withdrawn 2 cm and secured in place. The bronchoscopy further revealed copious old/clotted blood in the distal hypopharynx and between the wall of the ETT tube and lining of the trachea. Additionally, mild-to-moderate tracheobronchitis in the right mainstem bronchus and the left lung was found to be nearly completely occluded with a “cast” of old blood and mucus outlining the entire length of the left bronchus.

**Figure 1:**
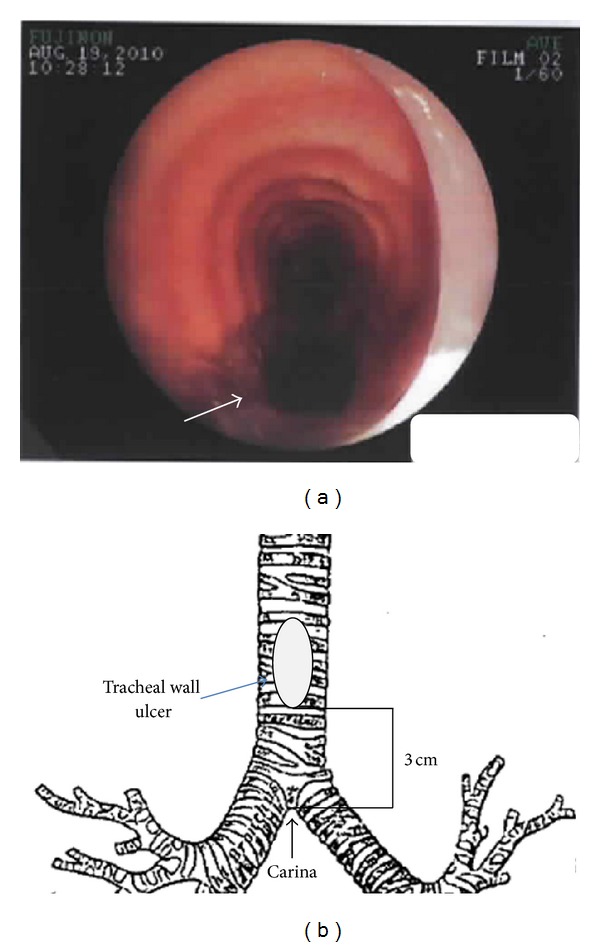
(a) Bronchoscopic visualization of the tracheal lesion (white arrow). (b) Position of posterior tracheal wall lesion in reference to the carina.

Four days later, our service was consulted for PCT placement. After confirmation of informed consent and patient identification, the patient was medicated with 200 mcg of fentanyl, 4 mg of midazolam, and 150 mg of rocuronium. A flexible fiberoptic bronchoscope was advanced into the ETT before starting the procedure; no abnormalities were observed. The ETT cuff was deflated and slowly moved directly above the vocal chords. A second bronchoscopic view revealed mild-to-moderate persistent nonpurulent tracheobronchitis and an ulcer on the posterior tracheal wall noted 3-4 cm above the carina extending 2 cm in length and 1 cm in width (Figure 1). Due to the length of the ulcer, obtaining a proper seal with the balloon of the Shiley tracheostomy tube was expected to be difficult. Therefore, the tracheostomy procedure was aborted, and the ENT service was consulted for evaluation. The ETT was changed to a reinforced ETT following bronchoscopic guidance. FiO_2_ was weaned from 100% to 40%, oxygen saturation was kept greater than 92%, respiratory rate was reduced to maintain a PaCO_2_ between 35 and 45 mmHg, and a bronchodilator treatment was recommended.

Later that evening the patient was transported to the operating room for an open tracheostomy by the ENT service. After the incision (approximately 2 cm below the cricoid cartilage) and dissection (superior to the thyroid isthmus), the second tracheal ring was identified. The ETT was removed just superior to the tracheal window under direct visualization with a flexible bronchoscope. The before-mentioned ulcer was identified inferior to the tracheal window and confirmed with its distal end 3 cm above the carina. Upon careful fiberoptic evaluation, the surgeon suspected the ulcer to be a tracheoesophageal (TE) fistula. At this time, the decision was made to place another reinforced ETT through the tracheal stoma, sutured to the skin, with the tip 0.5 cm above the carina and the cuff inferior to the TE fistula. The severity and location of the ulcer warranted an esophagogastroduodenoscopy to rule out a TE fistula. After evaluation with a rigid esophagoscope, a small amount of petechial type hemorrhage next to the tracheal ulcer, but no fistula, was identified.

Four days later, the ENT service replaced the reinforced ETT with a size six cuffed TracheoSoft XLT Extended-Length Tracheostomy Tube to bypass the ulceration. Over the following week, the patient was weaned from mechanical ventilation, determined stable, and discharged with the tracheostomy cannula sutured in place.

## 3. Discussion

The use of flexible bronchoscopy during PCT reduces the risk of procedural-related complications. Reported benefits include protection against loss of airway, submucosal flaps, improper positioning of the initial needle puncture, fractures of the anterior tracheal ring, misplacement of the tube, and discovery of tracheal stenosis and tracheomalacia [11]. Additionally, deflating the ETT cuff and withdrawing the ETT to a level above the vocal cords allow assessment of possible tracheal damage. Therefore, early recognition and treatment of a tracheal wall lesion is possible.

Intubations of one to five weeks can cause erosion and ischemic damage to the trachea, potentially yielding stenosis of the trachea or glottis, tracheomalacia, tracheoesophageal fistula, tracheal rupture, or ventilator-associated pneumonia [12]. These tracheal lesions are relatively common with the duration of intubation being the only independent risk factor [13]. ETT cuff inflation pressure greater than 30 cm H_2_O or that exceeding capillary perfusion pressure is also a risk factor for tracheal lesions [12].

The subglottic positioning of the artificial airway prevents proper visualization of tracheal lesions which can be fatal if undetected. Our initial fiberoptic passage through the ETT revealed no abnormalities or injuries because the ETT tip was 1 cm above the carina, and the bronchoscopy was focused on the acute respiratory deficiency in the left lung. The large tracheal ulcer was detected four days later, with the ETT withdrawn, exposing the distal trachea to direct visualization prior to the PCT. The likely cause of the ulcer was tracheal wall ischemia from over inflation of the ETT cuff; however, intracuff pressures were not recorded via a manometer throughout the hospital stay. The traumatic placement of the NG tube is excluded as the cause because endotracheal anesthesia was already initiated for the angiogram prior to its placement. If the patient's tracheal injury remained undetected during the initial assessment, blind placement of the PCT over the ulceration could have caused an iatrogenic esophageal rupture or perforation, and the PCT would have been the blamed cause.

Possible complications have been attributed to bronchoscopy such as increased risk for hypercarbia, respiratory acidosis, intracranial pressure, and loss of airway in some cases [14–16]. Additionally, bronchoscopy was observed to be cumbersome and sometimes unfeasible due to logistic reasons [17]. Despite this, in most situations the benefits of bronchoscopy before PCT likely outweigh the disadvantages.

Paran and colleagues developed a modified PCT technique: guidance provided by the user's finger [18]. Their modified technique was evaluated in 61 patients, three experienced complications. In response, Gründling et al. proposed that bronchoscopy could have prevented these complications, [19] and Melloni et al. recognized the unnecessary exposure of patients to potentially serious complications [20].

Our presented case reveals a critical example where devastating complications with a PCT were avoided after the usage of fiberoptic bronchoscopy inspection. Because of the increased likelihood of ischemic lesions after prolonged intubation, clinicians should routinely assess the trachea with a bronchoscope for unexpected tracheal pathology in addition to a constant visual survey during the PCT. We emphasize that bronchoscopy-guided PCT provides significant benefits and should be performed regardless of initial necessity.

## References

[1] Kornblith LZ, Burlew CC, Moore EE (2011). One thousand bedside percutaneous tracheostomies in the surgical intensive care unit: time to change the gold standard. *Journal of the American College of Surgeons*.

[2] Susarla SM, Peacock ZS, Alam HB (2012). Percutaneous dilatational tracheostomy: review of technique and evidence for its use. *Journal of Oral and Maxillofacial Surgery*.

[3] Ciaglia P, Firsching R, Syniec C (1985). Elective percutaneous dilatational tracheostomy: a new simple bedside procedure; preliminary report. *Chest*.

[4] Barba CA, Angood PB, Kauder DR (1995). Bronchoscopic guidance makes percutaneous tracheostomy a safe, cost- effective, and easy-to-teach procedure. *Surgery*.

[5] Winkler W-B, Karnik R, Seelmann O, Havlicek J, Slany J (1994). Bedside percutaneous dilational tracheostomy with endoscopic guidance: experience with 71 ICU patients. *Intensive Care Medicine*.

[6] Park H, Kent J, Joshi M (2013). Percutaneous versus open tracheostomy: comparison of procedures and surgical site infections. *Surgical Infections*.

[7] Fernandez L, Norwood S, Roettger R, Gass D, Wilkins H (1996). Bedside percutaneous tracheostomy with bronchoscopic guidance in critically ill patients. *Archives of Surgery*.

[8] Marelli D, Paul A, Manolidis S (1990). Endoscopic guided percutaneous tracheostomy: early results of a consecutive trial. *Journal of Trauma*.

[9] Jackson LSM, Davis JW, Kaups KL (2011). Percutaneous tracheostomy: to bronch or not to bronch-that is the question. *Journal of Trauma*.

[10] Dennis BM, Eckert MJ, Gunter OL, Morris JA, May AK (2013). Safety of bedside percutaneous tracheostomy in the critically ill: evaluation of more than 3,000 procedures. *Journal of the American College of Surgeons*.

[11] Marx WH, Ciaglia P, Graniero KD (1996). Some important details in the technique of percutaneous dilatational tracheostomy via the modified seldinger technique. *Chest*.

[12] Seegobin RD, van Hasselt GL (1984). Endotracheal cuff pressure and tracheal mucosal blood flow: endoscopic study of effects of four large volume cuffs. *British Medical Journal*.

[13] Touat L, Fournier C, Ramon P, Salleron J, Durocher A, Nseir S (2013). Intubation-related tracheal ischemic lesions: incidence, risk factors, and outcome. *Intensive Care Medicine*.

[14] Beiderlinden M, Walz MK, Sander A, Groeben H, Peters J (2002). Complications of bronchoscopically guided percutaneous dilational tracheostomy: beyond the learning curve. *Intensive Care Medicine*.

[15] Reilly PM, Sing RF, Giberson FA (1997). Hypercarbia during tracheostomy: a comparison of percutaneous endoscopic, percutaneous Doppler, and standard surgical tracheostomy. *Intensive Care Medicine*.

[16] Reilly PM, Anderson HL, Sing RF, Schwab CW, Bartlett RH (1995). An unrecognized phenomenon during percutaneous endoscopic tracheostomy. *Chest*.

[17] Maddali MM, Pratap M, Fahr J, Zarroug AW (2001). Percutaneous tracheostomy by guidewire dilating forceps technique: review of 98 patients. *Journal of Postgraduate Medicine*.

[18] Paran H, Butnaru G, Hass I, Afanasyv A, Gutman M (2004). Evaluation of a modified percutaneous tracheostomy technique without bronchoscopic guidance. *Chest*.

[19] Gründling M, Pavlovic D, Kühn S-O, Feyerherd F (2005). Is the method of modified percutaneous tracheostomy without bronchoscopic guidance really simple and safe?. *Chest*.

[20] Melloni G, Libretti L, Casiraghi M, Zannini P, Paran H, Gutman M (2005). A modified percutaneous tracheostomy technique without bronchoscopic guidance: a note of concern. *Chest*.

